# Cushing’s Syndrome due to a non-adrenal ectopic adrenocorticotropin-secreting ewing sarcoma in a child

**DOI:** 10.1186/1687-9856-2013-S1-P114

**Published:** 2013-10-03

**Authors:** Nancy G Honor

**Affiliations:** 1 University of the Philippines-Philippine General Hospital

## 

Ectopic ACTH Syndrome (EAS) is extremely rare in pediatric age group. Sarcomatous tumors causing EAS are even rarer. A nine year and 4 months old male Filipino presented with a 6 months history of gradually enlarging mass on the left distal thigh. He experienced increasing appetite, rapid weight gain of about 27kgs in 5 months, recent onset of widespread acne and darkening of skin around the neck. Physical Examinations revealed cushingoid face, widespread acne, facial hair, phlethora, buffalo hump, truncal obesity, purple abdominal striae and severe acanthosis nigricans on the neck and axilla. Non-inflamed mass and slight tenderness over the left distal thigh were noted. Patient was hypertensive. Weight & height for age were at Z score above 3 and -1, respectively. Body Mass Index at Z score above 2. His pubertal development was classified as Tanner stage 1 testicular and pubic hair development. Work ups revealed hypernatremia, hypokalemia, metabolic alkalosis and elevated levels of serum cortisol and ACTH. Low and high dose dexamethasone suppression tests failed to suppress serum and urine free cortisol levels, findings which were consistent with ectopic ACTH secreting tumor. Radiological findings were normal skull and chest roentgenograms and normal computed tomographic scan of chest and abdomen. Magnetic resonance imaging of Hypothalamo-pituitary was also normal. An Immunohistochemistry result of left distal thigh mass was consistent with Ewing Sarcoma. The patient received chemotherapy with Vincristine, Doxorubicin and Cyclophosphamide. Unfortunately, patient succumbed to death 5 days post chemotherapy due to severe sepsis.

